# Supplementary material for: Supporting newly graduated medical doctors in managing COVID-19: An evaluation of a Massive Open Online Course in a limited-resource setting
Source: PLoS One. 2021 Sep 10;16(9):e0257039. doi: 10.1371/journal.pone.0257039 (PMC8432880; doi:10.1371/journal.pone.0257039)
Supplement: Attachment — Submitted filename: MTPC PLoS ONE_Response to reviewers AF010721.docx [file pone.0257039.s001.docx]

**Point-by-point response to reviewers**

**“Supporting newly graduated medical doctors in managing COVID-19: An evaluation of Massive Open Online Course in a limited resource setting”**

**PONE-D-21-12216**

Dear Chief Editor and Reviewers of PLOS ONE,

On behalf of the authors, I would like to extend our appreciation and gratitude towards positive and encouraging feedback on our manuscript submission titled above. We have tried to revise the manuscript accordingly, with the following details:

| **No** | **Feedback** | **Revision** |
| --- | --- | --- |
|  | **General Feedback** |  |
| 1. | Please ensure that your manuscript meets PLOS ONE’s style requirements, including those for ﬁle naming. | Thank you. We have tried to fulfil the style requirements accordingly. |
| 2. | Please include additional information regarding the survey or questionnaire used in the study and ensure that you  have provided suﬃcient details that other could replicate the analyses. For instance, if you developed a questionnaire as part of this study and it is not under a copyright more restrictive than CC-BY, please include a copy, in both the original language and English, as Supporting Information. Moreover, please include more details on how the questionnaire was pre-tested, and whether it was validated. | We developed the questionnaire to describe the satisfaction of the participants towards the MOOC and to provide us further information on area for improvement., We have included the complete questionnaire items in Table 3.  We also add further information on the questionnaire in the Method section.  “…The questionnaire items were validated by the team, were pretested to medical students who completed another MOOC on Covid-19, and revised accordingly.” (Page 7, line 130-132). |
| 3. | In your Data Availability statement, you have not speciﬁed where the minimal data set underlying the results described in your manuscript can be found. PLOS deﬁnes a study's minimal data set as the underlying data used to reach the conclusions drawn in the manuscript and any additional data required to replicate the reported study ﬁndings in their entirety. All PLOS journals require that the minimal data set be made fully available. | The minimal dataset underlying the results can be accessed to the link below:  https://bit.ly/MTPC-I2021 |
| 4. | We note that Figure 2 your submission contains map images which may be copyrighted. All PLOS content is published under the Creative Commons Attribution License (CC BY 4.0), which means that the manuscript, images, and Supporting Information ﬁles will be freely available online, and any third party is permitted to access, download, copy, distribute, and use these materials in any way, even commercially, with proper attribution. For these reasons, we cannot publish  previously copyrighted maps or satellite images created using proprietary data, such as Google software (Google Maps,  Street View, and Earth). | We have changed the figure by using the map obtained from [**https://apps.nationalmap.gov/viewer/**](https://apps.nationalmap.gov/viewer/)  It is not a copyrighted material and can be used under Creative Commons Attribution License as requested. |
| 5. | Please upload a copy of Supporting Information Figures 1-2 and Table which you refer to in your text on page 24. | We have uploaded the supporting information of Figures 1 and 2 separately, as well as incorporated the figures in the main text. |
| 6. | Please review your reference list to ensure that it is complete and correct. If you have cited papers that have been  retracted, please include the rationale for doing so in the manuscript text, or remove these references and replace them with relevant current references. Any changes to the reference list should be mentioned in the rebuttal letter that  accompanies your revised manuscript. If you need to cite a retracted article, indicate the article’s retracted status in the references list and also include a citation and full reference for the retraction notice. | We have reviewed the reference list and the references are aligned with those referred in the text. |
|  | **Reviewer 1** |  |
| 1. | The manuscript is well drafted and highlights the strength of the MOOC for conducting courses on medical science. | Thank you very much for your positive comments on our manuscript. |
| 2. | Evaluation of the audience attentiveness should also be highlighted i.e. how attentive were the participants during online classes. | We have added further analysis aiming to assess the audience attentiveness by exploring the access time and duration in each section and by comparing them with the expected access time.  “The MOOC platform also allowed us to collect data in regards to access time of which participants completed each pre- and post-test as well as the number of attempts for each test. The data was then used to analyze the access duration of each section.” (Page 7, lines 132-134)  “The duration of participants’ access to each section was compared with the expected minimum duration estimated by course developers.” (Page 7, lines 144-146)  “We conducted access duration analysis of each section in attempt to evaluate users’ participation within the course. We also administered questionnaire on how participants perceived the course (at the end of Part A).” (Page 12, lines 244-246)  “We analyzed the duration of which participants accessed each section and compared it with the expected minimum duration estimated by course developers according to types of learning activities in each section. The result is shown in Table 1. The result showed that the duration of access was lower than the expected minimum duration estimated by course developers. It also showed that despite the additional sections in Batch 2, the average access duration of Batch 2 participants was shorter than Batch 1 participants.” (Page 13, lines 254-259)  Table 1. (Page 13)  We also added further details in the discussion on participants’ attentiveness in the MOOC, as follows:  “The limited use of learning activities that induced participation and interaction may have also played a role in the short average access duration among participants in both batches. The decreased average access duration of the course in Batch 2, despite the increase in learning sections, was also caused by the fact that the second batch took the online course after the pandemic had been going on for almost one year. Hence, some basic knowledge provided in the online course was already known from other open resources. This demonstrates the need for course providers to reprioritize and rapidly adjust the content regularly.” (Page 20, lines 370-377) |
| 3. | Time of viewing the online lecture should be incorporated for issuing the certiﬁcate of completion. | Thank you for your suggestion. We agree that time of viewing of the materials in the MOOC should be considered for the issue of certificate of completion since it shows attempts of the course provider to document the process. We haven’t done this in our current platform. However, we tried to employ the restriction feature which encourage or enforce participants to complete one section before moving to another. We do realize that the time of viewing should be incorporated and note this as further area for improvement for our MOOC platform.  We added this realization in the discussion, as follows:  “We also note the importance of optimizing the MTPC-I feature to document the participants’ learning processes in more details such as time of viewing in each section and further consider it in issuing certificate of completion in the future.” (Page 22, lines 418-420) |
| 4. | Both the ﬁgures provided are not readable. Improve the ﬁgure quality. | We replaced Figure 1 with larger image with the roadmaps from Batch 1 and 2 are placed in stack. We also used bigger fonts to make it more readable.  We used copyrighted map images from <https://apps.nationalmap.gov/viewer/>, and adopted it to replace previous image in Figure 2. We also used bigger fonts to make it more readable. |
|  | **Reviewer 2** |  |
|  | First, I would like to thank the authors for their initiative role to give a hand facing the COVID-19 pandemic. I checked the course website. It is very user-friendly, organized, and well-prepared.  Generally, the manuscript is clear and accepted, but there are a few questions and comments: | Thank you very much for the positive feedback. We have revised the manuscript accordingly. |
| 1. | How the authors justify the difference between the number of participants in the two batches? | The number of participants in the two batches is a given number from the MoH. Our team caters the need of the MoH to support essential knowledge of COVID-19 for the newly graduated medical doctors who are assigned to complete an internship program in the given period of time.  We emphasized the information as follows:  “Participants in the Indonesian National Internship Medical Doctors year 2020 were determined by the MoH and automatically enrolled in MTPC-I in two batches:   a. Batch 1, August 2020: 3,424 participants b. Batch 2, November 2020: 2,462 participants”   (Page 10, Lines 202-205) |
| 2. | Line 72-73: the sentence shows MOOC as if it just emerged during this pandemic, and it is not.  It should be rephrased. | Thank you for the correction remark. We agree that MOOC has emerged couple of years ago, and the use of MOOC has been increasing during this pandemic. Therefore, we rephrased the sentence as follows:  “Massive Open Online Course (MOOC) has been increasingly used as a timely platform during COVID-19 pandemic given a push to move learning activities online.[3]” (Page 4, lines 73-74) |
| 3. | Line 194: authors gave "unlimited opportunity".  Did you know how many opportunities were used for each section to be passed? I think this could indicate the diﬃculty of each section and refer to some potential improvements. | We added further information on this particular issue.  “The total attempts of the post-tests were also analyzed descriptively to compare the numbers across sections in the part A.” (Page 7, lines 146-147)  We also added the details in Table 2. |
| 4. | In the third theme (Line 288):  The authors did not report it related to Batch 2 as well. Didn't you receive any suggestions from this batch? | We have analyzed the open-ended questionnaires from Batch 1 and Batch 2, and we received many suggestions related to area of improvement from Batch 2 as well. One of the suggestions we received from Batch 2 is the need to add some information on how to manage COVID-19 in the rural areas and limited-resource setting, and we had mentioned it in Page 18 lines 318-320. For the revision of manuscript, we added a quotation related to the subject we mentioned above.  “*The module provides thorough knowledge related to COVID-19. It would be better if it also gives us [the interns] information on how to manage COVID-19 in rural areas or in a limited resource setting.” (NDP (F), Sumatera, batch 2)*” (Page 20, lines 329-331) |
| 5. | Line 321 - 327: Authors showed the critical needs they considered in THEIR work, but there is a reference at the end of the sentences!!  I cross-checked this reference, but I think it is not clear what they are referring to. | Thank you for the note. We have revised it accordingly, and removed the reference in-text and in the reference list. |
| 6. | There are very frequent grammar and punctuation mistakes, for example, line 37, 43, 44, 48, 55, 57, 66,68, 78,..... and  almost all over the manuscript.  It is recommended to have proofreading revision by a native English speaker. | Thank you for the feedback. We used a professional proofreading service to help us improve the English writing of the manuscript. |
| 7. | Few references are in different styles as in line 71,....  Also, some hyperlinks in the reference list are not working | We revised the in-text citation in line 71 as follows:  “Given that their competence does not necessarily cover adequate knowledge on COVID-19 pandemic and comprehensive management,[2] attempts to support their knowledge are necessary.” (Page 4, lines 70-72)  We also checked the hyperlinks in the reference list, and made revision as follows:   - Hyperlinks in references number 3 and 19 were unable to open, so we remove the hyperlinks.   “3. Bylieva, D., Bekirogullari, Z., Lobatyuk, V., & Nam, T. Analysis of the consequences of the transition to online learning on the example of MOOC philosophy during the COVID-19 pandemic. *Humanities & Social Sciences Reviews*. 2020;8(4):1083-1093.” (Page 26, lines 476-478)  “19. AlQudah AA. Accepting Moodle By Academic Staff at The University of Jordan: Applying and Extending TAM In Technical Support Factors. *European Scientific Journal*, ESJ. 2014;10(18).” (Page 27, lines 521-523)   - We replaced the hyperlink in the reference list number 8, since the previous one was unable to open.   “8. Stracke CM, Tan E, Texeira A, et al. *Quality Reference Framework (QRF) for the Quality of Massive Open Online Courses (MOOCs)*. 2018. Retrieved from: [http://mooc-quality.eu/wp-content/uploads/2019/11/ Quality_Reference_Framework_for_MOOCs_v11.pdf](http://mooc-quality.eu/wp-content/uploads/2019/11/%20Quality_Reference_Framework_for_MOOCs_v11.pdf).” (Page 25, lines 489-490 and page 26, lines 491-492)   - We added hyperlink to the reference list number 23.   “22. Susanto AP, Findyartini A, Taher A, et al. COVID-19 in Indonesia: Challenges and Multidisciplinary Perspectives for a Safe and Productive New Normal. *Acta Med Indones*. 2020;52(4):423-430. Retrieved from: [http://www.actamedindones.org/index.php/ ijim/article/view/1594/pdf](http://www.actamedindones.org/index.php/%20ijim/article/view/1594/pdf).” (Page 27, lines 530-533) |

Thank you very much for your kind attention. We hope that the revisions are as expected, and the manuscript of our study can finally be published on the PLOS ONE.

Best regards,

Ardi Findyartini
